# What's New Is Old: Resolving the Identity of *Leptothrix ochracea* Using Single Cell Genomics, Pyrosequencing and FISH

**DOI:** 10.1371/journal.pone.0017769

**Published:** 2011-03-17

**Authors:** Emily J. Fleming, Amy E. Langdon, Manuel Martinez-Garcia, Ramunas Stepanauskas, Nicole J. Poulton, E. Dashiell P. Masland, David Emerson

**Affiliations:** 1 Bigelow Laboratory for Ocean Sciences, West Boothbay Harbor, Maine, United States of America; 2 Department of Biology, Swarthmore College, Swarthmore, Pennsylvania, United States of America; Netherlands Institute of Ecology, Netherlands

## Abstract

*Leptothrix ochracea* is a common inhabitant of freshwater iron seeps and iron-rich wetlands. Its defining characteristic is copious production of extracellular sheaths encrusted with iron oxyhydroxides. Surprisingly, over 90% of these sheaths are empty, hence, what appears to be an abundant population of iron-oxidizing bacteria, consists of relatively few cells. Because *L. ochracea* has proven difficult to cultivate, its identification is based solely on habitat preference and morphology. We utilized cultivation-independent techniques to resolve this long-standing enigma. By selecting the actively growing edge of a *Leptothrix*-containing iron mat, a conventional SSU rRNA gene clone library was obtained that had 29 clones (42% of the total library) related to the *Leptothrix*/*Sphaerotilus* group (≤96% identical to cultured representatives). A pyrotagged library of the V4 hypervariable region constructed from the bulk mat showed that 7.2% of the total sequences also belonged to the *Leptothrix*/*Sphaerotilus* group. Sorting of individual *L*. *ochracea* sheaths, followed by whole genome amplification (WGA) and PCR identified a SSU rRNA sequence that clustered closely with the putative *Leptothrix* clones and pyrotags. Using these data, a fluorescence *in*-*situ* hybridization (FISH) probe, Lepto175, was designed that bound to ensheathed cells. Quantitative use of this probe demonstrated that up to 35% of microbial cells in an actively accreting iron mat were *L*. *ochracea*. The SSU rRNA gene of *L*. *ochracea* shares 96% homology with its closet cultivated relative, *L*. *cholodnii*, This establishes that *L*. *ochracea* is indeed related to this group of morphologically similar, filamentous, sheathed microorganisms.

## Introduction

*L*. *ochracea* is a member of the neutrophilic, freshwater iron-oxidizing bacteria (FeOB), a group that accelerates Fe(II) oxidation in chalybeate waters and forms visible ocherous mats. In addition to playing an important role in the iron cycle, these microbial mats accrete rapidly and may decrease water flow, and sorb nutrients [1]–[3]. The FeOB are considered nuisance organisms when they cause biofouling of water distribution pipelines, or influence biocorrosion [4], [5]. In Europe, they have been recognized to have a beneficial role in water filtration systems due to the sorptive capacities of the Fe-oxyhydroxides [6], [7].

*L*. *ochracea* is often the most morphologically conspicuous member of FeOB communities due to its copious production of 1–2 micron wide smooth filamentous sheaths that are mostly devoid of cells [1], [8]–[10]. *L. ochracea* was first described in 1888 by Winogradsky [9], who identified the organism based on its distinctive sheath morphology. In the early days of light microscopy, it was natural to believe that these filamentous sheaths were synonymous with the abundance of *L*. *ochracea* cells [11]. Subsequent studies using epifluorescence microscopy coupled with nucleic acid binding dyes revealed the majority of sheaths were empty remains left by *L*. *ochracea* as it grew and oxidized Fe(II) [12].

Following the description of *L*. *ochracea,* other species of *Leptothrix* have been described along with a related genus, *Sphaerotilus*. The classification of these organisms was based on sheath production as well as Fe and/or Mn oxidation that resulted in metal impregnated sheaths [13]. Phylogenetic analysis has confirmed that these morphologically similar organisms are indeed close relatives, clustering together in the order *Burkholderiales* of the *Betaproteobacteria* [14]–[16]. Species in this cluster, e.g. *L*. *cholodnii*, *L*. *discophora*, *S*. *natans,* have been isolated and shown to be heterotrophs able to grow on a variety of carbon sources [10]. They may oxidize Fe(II) or Mn(II), and in the case of *L. discophora* Mn and Fe-oxidizing proteins have been described [17], [18]; however they do not require, beyond trace amounts, Fe or Mn for growth [13], [19]. Because of historical precedence, *L*. *ochracea* has remained the type species of the *Leptothrix* genus [20]. However, its taxonomic affiliation is based solely on morphological traits of cell shape and sheath formation, along with Fe-oxidation. Subsequent attempts to clarify *L*. *ochracea's* taxonomic affiliation and determine its physiology have generated more controversy than clarity [21].

We set out to determine the molecular phylogeny of *L*. *ochracea* by taking advantage of knowledge about its natural history and combining this with cultivation independent approaches. First, we characterized the microbial composition in an active freshwater Fe-oxidizing mat using a combination of SSU rDNA clone libraries, pyrotags, and DNA sequencing of individual cell filaments. We used this information to design a fluorescent *in situ* hybridization (FISH) probe putatively targeting *L*. *ochracea.* Hybridization of this probe to cells inside *L*. *ochracea* sheaths confirmed that the obtained SSU rRNA sequences were indeed of *L*. *ochracea,* enabling a detailed phylogenetic analysis.

## Methods

### Characterization of a freshwater iron seep and measurement of physiochemical parameters

All environmental samples were collected from flocculant iron mats in a soft-water, first-order ephemeral, intermittent stream located near Lakeside Drive (LD) in Boothbay Harbor, ME (43°51.699′N 69°38.929′W) during August 2008 and July 2009. The site was located two miles from our laboratory, and was visited on a weekly and even daily basis. Routine measurements were taken for water temperature, pH (Oakton 110 series meter), and Fe(II) concentrations (determined by the ferrozine method; [22]). Oxygen in mats was measured by ISO_2_ Oxygen meter and a OXELP probe (World Precision Instruments, Sarasota FL). For the cultivation-independent studies, samples were collected from mats that had formed within the previous 24 hrs. To confirm these mat samples were active, they were examined by phase contrast and epifluorescence microscopy with an Olympus BX60 microscope (Center Valley, PA). Samples retained for analysis were dominated by characteristic smooth microtubular sheaths of *L*. *ochracea*, and approximately 10% of the sheaths contained cells.

Several attempts were made to isolate *Leptothrix* or *Sphaerotilus* from LD mat samples using either *Sphaerotilus* medium [23] or agar plates with autoclaved sterilized LD waters and agarose (1.5% final concentration).

### SSU rDNA gene V4 region pyrosequencing library

A tagged pyrosequencing library of the hypervariable V4 region was generated from frozen LD bulk mat samples. The frozen mat was thawed, concentrated in an Eppendorf centrifuge (4000 x g) and resuspended in 20 mM phosphate buffer (pH 7.4). This phosphate wash was repeated twice prior to extracting the DNA using the MoBio Soil DNA extraction kit. Extracted DNA was sent to the Michigan State University sequencing facility (http://rtsf.msu.edu/) for pyrosequencing using the FLX System (454 Life Sciences, Branford, CT, USA) with V4 primers (**Table 1**) to target the V4 hypervariable region of SSU rRNA gene.

**Table 1:** Probes and primers used in this study.

Probe/primer	Sequence (5′–3′)	Reference
EUB338	cy3 gct gcc tcc cgt agg agt	63
NON338	cy3 act cct acg gga ggc agc	64
BET 42a	cy3 gcc ttc cca ctt cgt tt	38
GAM42a	Fl gcc ttc cca cat cgt tt	38
PS-1	cy3 gat tgc tcc tct acc gt	39
Lepto 175	cy3 atc cac aga tca cat gcg	this study
V4-F	ayt ggg ydt aaa gng	65
V4-R1	tac nvg ggt atc taa tcc	65
V4-R2	tac crg ggt htc taa tcc	65
V4-R3	tac cag agt atc taa ttc	65
V4-R4	tac dsr ggt mtc taa tcn	65
27F	agr gtt yga tym tgg ctc ag	65
907R	ccg tca att cmt ttr agt tt	65
1492R	tac ggy tac ctt gtt acg act t	66
gyrB F	kcg caa gcg scc sgg cat gta	67
gyrB R	ccg tcs acg tcg gcr tcg gtc at	67

### SSU rDNA clone library

To select for active *L*. *ochracea*, freshly precipitated mat samples (15 mL) were collected from the edges of the actively growing microbial mat with a sterile pipette. To enrich ensheathed cells and decrease the number of planktonic cells, the samples were filtered through a sterile 8 µm mesh filter (Biodesign Inc. of New York) mounted on a 25 mm glass frit support (VWR) using a vacuum pump. The DNA was extracted, amplified, cloned and sequenced using standard methods and is detailed in **File S1.**

### Single cell genomic analysis

Single cell sorting, whole genome amplification (WGA) and PCR-based analyses were performed at the Bigelow Laboratory Single Cell Genomics Center (www.bigelow.org/scgc). Iron mat samples were sheared by vortexing between 10 and 60 seconds, stained with 5 µM SYTO-9 nucleic acid stain (Invitrogen) and passed through a 70 µm mesh-size cell strainer (BD). Using a MoFlo™(Beckman Coulter) flow cytometer equipped with a 70 µm nozzle orifice and using a 488 nm laser excitation, fluorescence activated cell sorting (FACS) from LD iron mat samples was done to identify a sort region that would yield ensheathed *L*. *ochracea* cells. The cytometer was triggered on SYTO-9 fluorescence (green), and between 1000–2000 particles were collected from different gated regions for microscopic observation. The presence of ensheathed *L*. *ochracea* cells was confirmed from a gated region using phase contrast and epifluorescence microscopy (**Figure S1**).

For downstream DNA analyses, individual target particles were sorted using side scatter trigger and “purify 0.5 drop” mode and deposited into 384-well PCR microplates containing 600 nL per well of the prepGEM Bacteria (ZyGEM) reaction mix. The microplates were then stored at −80°C until further processing. Of the 384 wells, 311 were dedicated for single sheath particles, 62 were used as negative controls (no droplet deposition), 3 wells received 10 sheath particles each (positive control type 1) and 8 wells received 40 fg human DNA (Promega, positive control type 2). Prior to single cell sorting, the cytometer was cleaned thoroughly with 10% bleach. A 1% NaCl solution (0.2 µm filtered and UV treated) was used as sheath fluid. All tubes, plates and reagents were UV treated to remove DNA contamination. Full details on cleaning methods and instrument preparation are described previously in Stepanauskas and Sieracki [24].

To lyse the cells, microplates with the sorted material were incubated at 37°C and 75°C, 20 min each. This sequentially activated lysozyme and the prepGEM protease. After the enzymatic treatment, cold KOH was used to ensure complete cell lysis and to denature DNA [25]. Genomic DNA from the lysed cells was then amplified using multiple displacement amplification (MDA) [26] in 10 µL final volume, using Repliphi (Epicenter) polymerase and buffers and phosphorylated random hexamers (NNNN*N*N; IDT). The MDA reactions were run at 30°C for 16 h, then inactivated by a 10 min incubation at 65°C. The amplified genomic DNA was stored at −80°C until further processing. We refer to the MDA products originating from individual cells as single amplified genomes (SAGs).

The MDA products were screened for the presence of the bacterial SSU rRNA gene by PCR, using 500 nL of 50-fold diluted WGA products as template in a 5 µL real-time PCR with primers 27F and 907R (**Table 1**) (Integrated DNA Technologies, IDT). Real-time PCR was performed on a LightCycler 480 using SYBR Green I master mix (Roche). We used the real-time PCR kinetics and melting curve analysis to detect successful reactions, the amplicons of which were sequenced from both ends by Beckman Coulter Genomics facility (http://www.beckmangenomics.com/genomic_services/sample_submission.html) using Sanger technology.

### Sequence analysis

The SSU rRNA gene sequences from SAGs and clones were assembled and manually curated using Geneious (Biomatters Ltd. Aukland, New Zealand) or Sequencer (Gene Codes Corporation, Ann Arbor, MI) software. The consensus sequences were aligned using the Silva aligner (www.arb-silva.de, March 23, 2010). The phylogeny was determined with the ARB program and RAxML (Neighbor Joining tree; [27] Maximum Liklihood; [28]).

High-quality pyrosequencing reads were identified using PyroTagger [26]. Using the BLAST 2.2.22+ program (ftp://ftp.ncbi.nlm.nih.gov/blast/), we identified reads that were ≥99% identical to the V4 region in the putative *L. ochracea* clones and SAGs. These closely related sequences were then aligned using MUSCLE [30] within the Geneious software and evolutionary trees were constructed in ARB. The analysis of SSU rRNA gene sequences is detailed in **File S1**.

The following sequences were submitted to GenBank: the complete SSU rRNA gene sequence from *L*. *ochracea* clones (HQ317218-HQ317231), the complete SSU rDNA gene sequences from single amplified genome AAA018-L12 (HQ290516), the partial gene sequence from the *L*. *ochracea* gyrB gene sequence (HQ290424) the non-chimeric partial SSU rRNA gene sequence clone libraries (HQ290425-HQ290476) and the partial SSU rRNA gene sequences from the single amplified genomes (HQ290477-HQ290515). The cleaned pyrosequencing hypervariable V4 region reads were submitted to the NCBI short read archive(http://trace.ncbi.nlm.nih.gov/Traces/sra/sra.cgi?) under the accession number (SRP005891).

### FISH analysis

For FISH, mat samples from either LD, or cultivated control strains were fixed at 4°C in 2.5% paraformaldehyde (final conc.; per Fuchs et al., [31]) for 90 min. After fixation samples were rinsed twice with PBS via centrifugation at 2150 x g, resuspended in 1∶1 PBS:Molecular Biology Grade Ethanol, Sigma and stored at −20°C until use. A FISH probe for *L*. *ochracea* (Lepto175; **Table 1**) was designed using the SSU rRNA gene sequences from the clone and SAG libraries and the ARB software. The probe development and hybridizations were performed using standard methods [31] and are detailed in **File S1**. We found that iron oxyhydroxides interfere with FISH. To reduce this interference, the sample was dried on ClearCell slides with 7 mm diameter wells (ER-279W-2; ThermoScientific), not dehydrated with an ethanol series, and prehybridized with the blocking reagents typically used in CARD-FISH [32].

### Cell counts and Microscopy

Phase contrast and fluorescence microscopy were done using an epifluoresence Olympus BX60 microscope equipped with a QICAM Fast CTD camera (Qimaging, Surrey BC).

The percentage of *L. ochracea* cells in freshly formed LD mats was determined by FISH using six samples collected in 2008 or 2009 and are detailed in **File S1**. To determine the cell width and length, and sheath width of *L*. *ochracea* cells, and the total percent of sheaths filled with cells, photomicrographs were obtained from gluteraldehyde preserved samples, and analyzed using the ImageJ (http://rsbweb.nih.gov/ij/ [33]) plot profile tool.

Scanning Electron Microscopy of *L*. *ochracea* sheaths was done on freshly precipitated *L*. *ochracea*-rich Fe-mats using a Hitachi S-4700 FESEM at DBI Bioimaging (http://www.dbi.udel.edu/bioimaging/fesem.html) at the University of Delaware.

## Results

### Physicochemical parameters of LD Fe-mats and identification of *L*. *ochracea*

**Figure 1:**
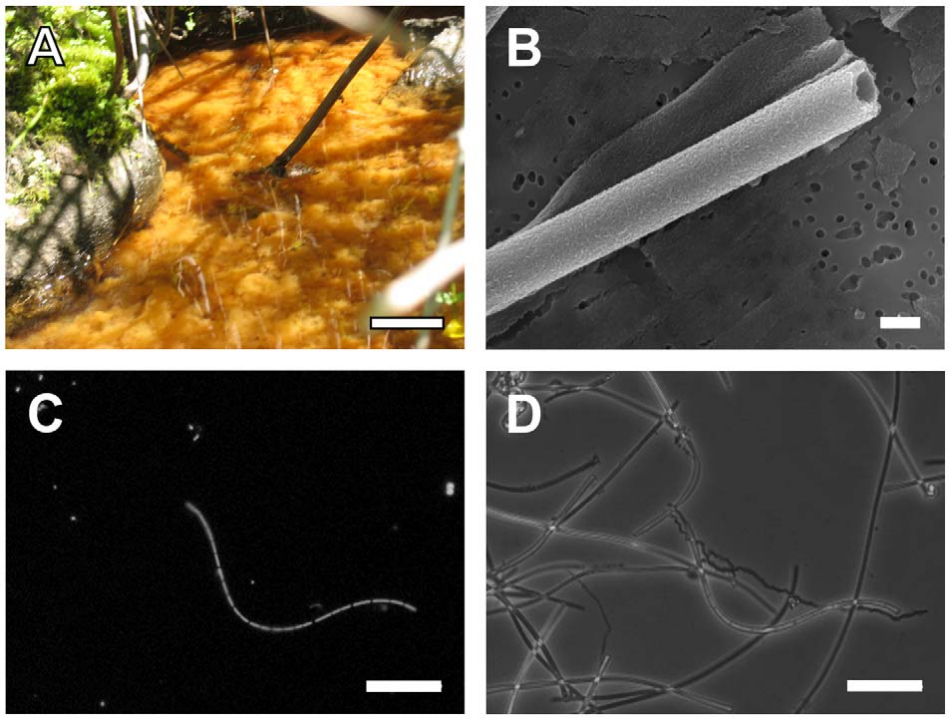
Photo-montage of *L*. *ochracea* sheaths and cells from Lakeside Drive. Summer time bloom of typical LD iron mats (A), Scanning electron micrograph of a typical *L. ochracea* sheath (B). Epi-fluorescence of Syto 13 stained cells (C) and phase contrast image (D) of filamentous cells and sheaths from LD iron mats. Scale bars are 10 cm, 10 µm, 10 µm and 10 µm, respectively.

The freshly precipitated iron mats in the stream on LD were used as the source material for identification and characterization of *L*. *ochracea*. The mats at LD consisted of a loose floc of Fe-oxyhydroxides that at times filled the entire depth of the 15 cm deep water column (**Figure 1A**), and were most abundant during June, July, and August. The presence of Fe-mats was sensitive to large rainfall events (>0.5 cm day^−1^) which flushed the accumulated iron mat downstream; however once normal flow rates (<3 cm s^−1^) resumed, and so long as Fe(II) concentrations were high (> 70 µM) the mats reestablished rapidly, typically in less than a week. During August 2008 and July 2009, when samples were taken for the analyses reported here, the water was consistently 15°C–17°C, the pH 6.0–6.5 and the Fe(II) 70 µM–160 µM. The stream was approximately half-saturated with O_2_ (approximately 150 µM at 15°C) and profiles within the mats revealed a gradual decrease in O_2_ concentration with depth. This confirmed previous reports of low O_2_ demand in this type of iron mats [34], [35]. Smooth tubular iron oxide encrusted sheaths formed the primary mat matrix. Based on observations by light microscopy, it was noted that samples collected at the edges of the mat contained more sheathed cells than deeper layers, and that the sheaths appeared less mineralized.

The putative *L*. *ochracea* sheathed cells (**Figure 1B & 1C**) were 1.0 µm ± 0.14 (σ, n = 32) wide and 3.04 µM ± 0.92 (σ, n = 23) long. The microtubular sheaths that encased the cells were free of unstructured particulate oxides (**Figure 1D** **)** and measured 1.1 µM ± 0.95 (σ, n = 15) wide. We estimated that 9.4% ± 3.32 (σ, n = 9 fields) of the sheaths in actively growing mat were filled with cells. Attempts to enrich and isolate heterotrophic *Leptothrix spp.* or *Sphaerotilus spp.* from LD mats did not indicate they were abundant. Plating mat samples on *Sphaerotilus* agar and LD waters yielded very few filamentous bacterial colonies that matched the morphological description of *Leptothrix* or *Sphaerotilus* (only 2 colonies present at the 100x dilution), indicating these heterotrophs were not abundant.

### Microbial diversity in the iron mats

**Figure 2:**
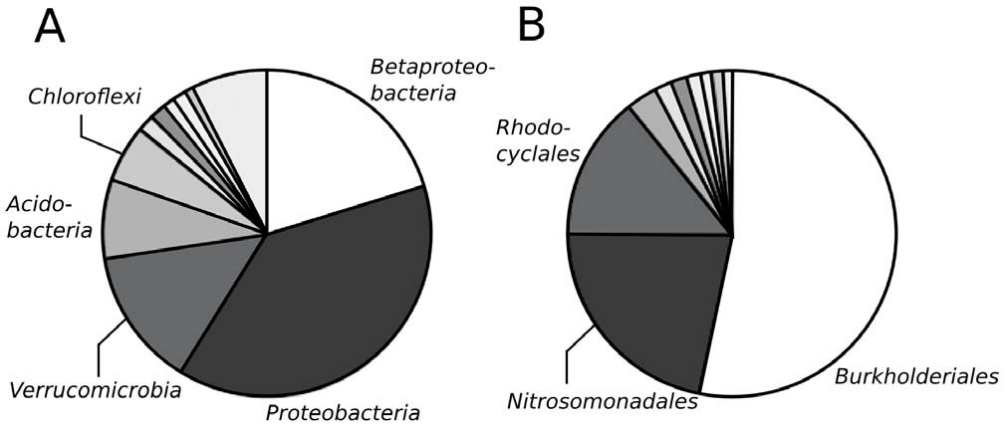
Bacterial composition in the bulk samples of Fe-oxidizing mats as determined by pyrosequencing of the SSU rDNA V4 region. Fraction of the various bacterial phyla (A), as determined by the RDP Classifier with ≥80% confidence; the unlabeled sections are (clockwise, starting after *Chloroflexi*) *Gemmatimonadetes*, unclassified, WS3, ZB2 and *Chlamydiae* and phyla that form less than 1% of the total sequences. A subset of the data that breaks out the different orders of *Betaproteobacteria* (B), as determined by alignment and parsimony analysis with the Silva database (release 102); the respective unlabeled orders are (clockwise), staring after *Rhodocyclales* unassigned, SCI-84, *Neisseriales*, UCT N117, *Hydrogenophilales*, 070125-BRIC7-5 and TRA3-20.

After processing and filtering the pyrosequencing library, a total of 9864 reads out of 16786 (59%) were deemed to be of suitable quality for phylogenetic analysis. In total, 8607 sequences that could be assigned to an OTU (an OTU is defined as sequences that have ≥97% identity) that contained ≥ 2 members, the remaining 1257 sequences were singletons. Overall, the largest number of OTUs (222) and greatest proportion of sequences (19.8%) were affiliated with the *Betaproteobacteria* (**Figure 2**). The *Burkholderiales* order and the *Comamonadaceae* family which include the cultivated *Leptothrix* and *Sphaerotilus* species and contained 10.3% and 9.6% of the total sequences, respectively. The OTU that was most closely affiliated with *Leptothrix* spp. contained 667 sequences. Representatives from *Gallionellaceae* (including *Sideroxydans*, *Gallionella*), that include freshwater, lithotrophic iron-oxidizing bacteria, were 2.5% of the sequences, and bacterial groups known to include Fe-reducers, e.g. *Geobacter*, *Rhodoferax*, *Ferribacterium*, and *Pelobacter,* were 6.1% of the sequences. Several other abundant OTUs (containing >100 sequences each) were affiliated with the *Gammaproteobacteria* the *Verrucomicrobia*, and the *Alphaproteobacteria*.

The conventional clone library contained a total of 69 sequences after quality control processing, and 41% of the total sequences, (**Figure S2**) that were ≥ 99% similar to one another and were affiliated with the *Betaproteobacteria*. When the phylogeny of this group was analyzed by distance algorithms it was clearly affiliated with the Burkholderiales family with its closest relatives being in the *Leptothrix*/*Sphaerotilus* group; but still distinct (≤96% identity) from the cultivated members of the *Leptothrix* and S*phaerotilus* genera. Other sequence groupings from the SSU rRNA gene clone library were far less abundant. Therefore, it was concluded that the most abundant sequence cluster represented putative clones of *L*. *ochracea*.

### Single cell analysis

**Figure 3:**
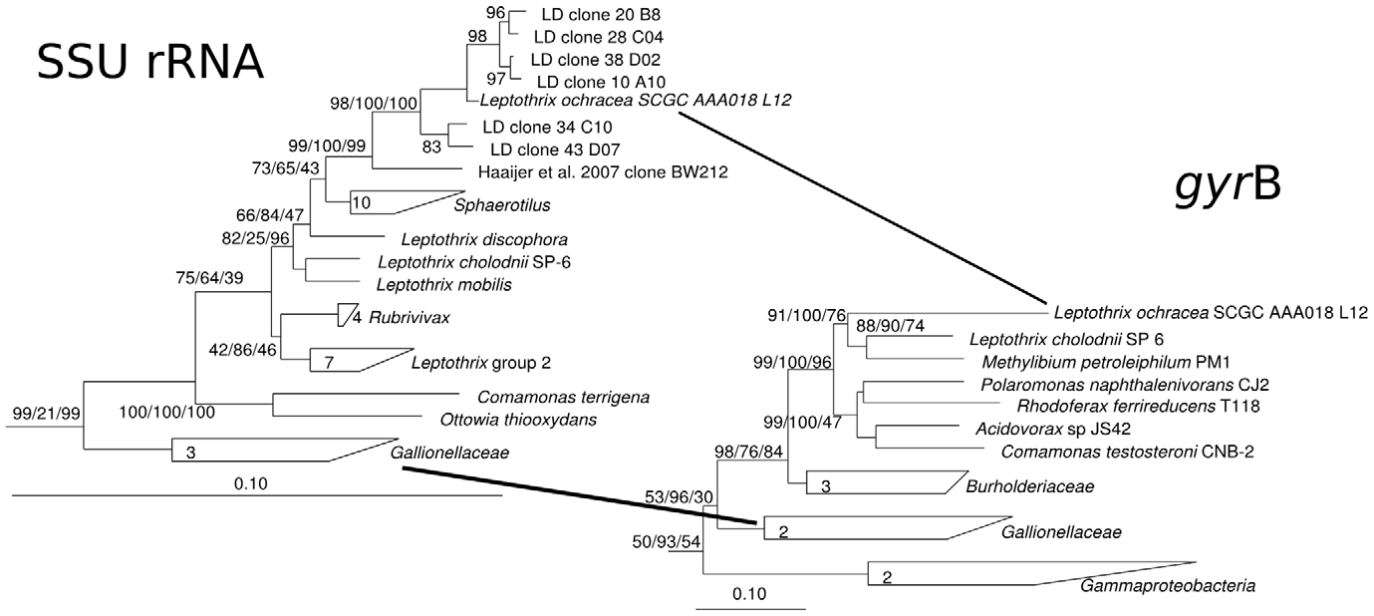
Phylogenetic tree of the SSU rRNA and *gyrB* genes from *Leptothrix* and several type species from the *Betaproteobacteria* as determined by Neighbor-Joining, with Jukes-Canter correction. Node percentages represent the bootstrap values from Neighbor Joining, Maximum Liklihood (RAxML with a Gamma distribution mode) and Parsimony (with phylip) methods. The tree shown is the most-likely tree from 1000 replicate trees with *Sulfitobacter guttiformis* (Y16427) as the outgroup.

The phylogenetic diversity of SAGs was similar to the diversity of the SSU rRNA gene clone library and the pyrosequencing data set (**Figure S2**), and contained representatives of genera known to oxidize iron or reduce iron. One SAG (SCGC AAA018-L12) was ≥99% identical to the putative *L*. *ochracea* SSU rRNA clones and >97% identical to only 2 sequences in GenBank (>99% for AY947983; [36], and 97% for EF079081; [37]). Furthermore it was only 95% similar to *Leptothrix cholodnii* SP-6, its closest relative among the cultured *Leptothrix*/*Sphaerotilus* group. A Maximum Likelihood phylogeny with other taxa in the *Betaproteobacteria* grouped the full-length sequence from the putative *L*. *ochracea* SAG and several clones from LD with the members of the *Leptothrix*/*Sphaerotilus* group (**Figure 3**). Phylogenic analysis of the *gyrB* gene sequence from the *L*. *ochracea* SAG also clustered with the gyrB gene sequence of *L*. *cholodnii* SP-6. A similarity matrix analysis indicated that the putative *L*. *ochracea* SAG SSU rRNA gene was distinctly different from all cultivated *Leptothrix*/*Sphaerotilus* species, with its closest relative being *L. cholodnii* strain SP-6 (96.3% identical). For all downstream *in silico* analysis and development of FISH probes, this SAG sequence was used as the putative *L*. *ochracea* SSU rRNA gene sequence.

### Enumeration of L. ochracea with fluorescence in situ hybridization (FISH)

**Figure 4:**
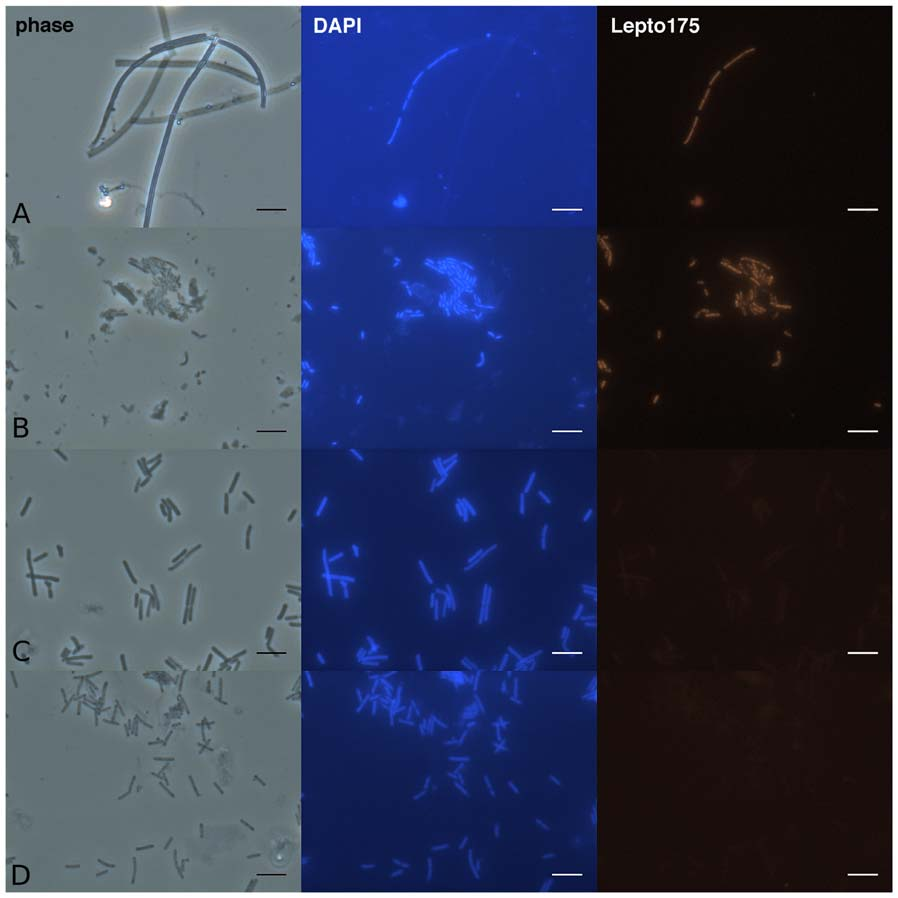
Micrographs of LD iron-mats (row A), *O*. *thiooyxgens*, (row B), *S. natans* (row C) and *L*. *cholodnii* SP-6 (row D) viewed with phase contrast, epi-florescence stained with DAPI, and epi-florescence with the Lepto175 probe applied. Scale bar is 5 µm. Cells were hybridized with the Lepto175 probe (hybridized at a 15% formamide concentration).

To confirm the phylogenetic affiliation of *L*. *ochracea* and link it to its recognized morphology, a FISH probe for the SSU rRNA gene was developed based on the sequence information gathered above. Initial results showed that the general *Betaproteobacteria* probe BET 42a bound to *L*. *ochracea* cells when used with GAM 42a as a competitor probe [38]. Furthermore, a *Leptothrix* specific probe (PS-1), designed to target *L*. *cholodnii* SP-6 [39] bound to *L*. *ochracea*, but only at low stringency (<5% formamide). Sequence comparison of the PS-1 probe and the putative *L*. *ochracea* SSU rRNA gene sequence showed a 2 bp mismatch (**Table** S**1**). Therefore, the Lepto175 probe was designed to specifically target the putative *L*. *ochracea* sequence. The probe sequence exactly matched 3 environmental sequences (AY947983, AM778004, and EF079081; [36], [37]) and the isolate *Ottowia thiooxydans* (AJ537466;[40]), a nonsheath-forming unicellular bacterium that is not known to oxidize Fe(II) match or hybridize with the PS-1 probe (**Table S1**). Under stringent conditions (up to 20% formamide; **Figure S3**) the probe bound well to ensheathed *Leptothrix ochracea* cells, (**Figure 4** **)**. This probe did not bind to *S*. *natans* (5 bp mismatch), or to *L*. *cholodnii* (4 bp mismatch) under stringent conditions (15% formamide) (**Figure 4**).

To assess natural populations of *L*. *ochracea* mats, were collected from the periphery of the LD mat from different days and years. Ensheathed cells that bound to the Lepto175 probe composed 35–85% (n = 7) of the total bacterial population able to hybridize with universal bacterial probe EUB 338, and up to 32% (n = 6) of the total microbial population stained by the DNA dye Syto13. Cells that hybridized to the Lepto175 probe were all ensheathed and only filled approximately 10% of the sheaths present. These observations showed that the cell morphology was consistent with the historical description of *L*. *ochracea*.

### Microdiversity analysis

**Figure 5:**
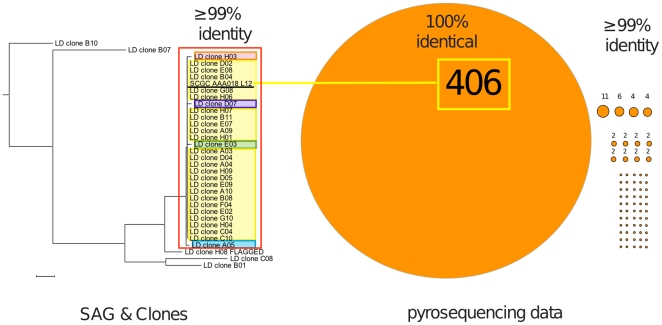
Microdiversity of *L*. *ochracea* clones and pyrosequencing reads. The Neighbor-Joining tree was constructed using the Jukes-Cantor substitution model with sequences of the hypervariable region V4 of the SSU rRNA gene from SAG and clones that matched the Lepto175 probe. Ribotypes boxed in red had ≥ 99% identity to one another and each highlighted clone(s) represent a unique ribotype in the clone library. Each circle or dot represents a unique ribotype of *L*. *ochracea* found in the pyrosequencing library that was either ≥100 or 99% identical to *L*. *ochracea* SAG and clones boxed in yellow.

To further understand the population structure of *L*. *ochracea* within these iron mats, we analyzed the microdiversity of sequences obtained both through clone libraries and pyrosequencing. To do this, a custom database was built from the tagged pyrosequences and then subjected to BLASTn similarity searches using the putative *L. ochracea* sequences from SAG and clone libraries (see details in Material and Methods). From these, 501 related sequences (5% of the total reads) that were ≥ 99% similar to the putative *L. ochracea* clones and the SAG were used to define the microdiversity of *L*. *ochracea* ribotypes. Clustering analysis of highly similar pyrosequencing reads suggested that 67 unique ribotypes were present (**Figure 5**). Remarkably, the dominant ribotype found in the pyrosequencing data set (406 reads) was identical to the hypervariable region V4 of the several clones and the SAG SCGC AAA018-L12. This suggests that the cell or filament of cells isolated by FACS from the Fe-mats was probably the most abundant and dominant *L*. *ochracea* ribotype in the environment. Other putative *L*. *ochracea* ribotypes (those with ≥99% to the SAG) represented only 18% of the *L*. *ochracea* pyrosequencing reads and were not identical to any of the sequences in the clone library.

## Discussion

Through the use of SSU rRNA gene analysis, WGA of single cells, and specific identification of cells in environmental samples using FISH we have confirmed that *L. ochracea* is phylogenetically related to other species of *Leptothrix* and *Sphaerotilus.* The results presented here settle a 120 year old mystery concerning the provenance of *L*. *ochracea* [20], [21]. In this case, the common traits of the ensheathed filamentous cells, the capacity to oxidize Fe(II), and the phylogeny are linked in the *Leptothrix*/*Sphaerotilus* group. The phylogenetic analyses, based on the SSU rRNA gene sequences of one *L. ochracea* SAG, and several clones, show that *L*. *ochracea* forms a unique cluster within this group (**Figure 3**). Among the known species of *Leptothrix* and *Sphaerotilus*, its closest relative is *L*. *chlodonii*. However, the different treeing methods used to generate both the SSU rRNA gene phylogeny and the *gyrB* gene phylogeny (Maximum Liklihood, Neighbor Joining, Parsimony) indicate that *L*. *ochracea* is related equi-distant from known *Leptothrix* and *Sphaerotilus* species. Based on this phylogenetic analysis, it is reasonable to conclude that *L ochracea* is a relative of *Leptothrix* and *Sphaerotilus* spp., but that it represents a unique lineage, which is consistent with its apparent differences in morphology and habitat.

### Ecological implications

Given the apparent differences in physiology among the cultivated members of the *Leptothrix*/*Sphaerotilus* cluster, it was not obvious that *L*. *ochracea* would share a close phylogenetic relationship with other *Leptothrix* and *Sphaerotilus* spp. Both sheath formation and Fe-oxidation are properties found throughout the bacterial tree of life [15], [41]–[45]. Our cultivation-based attempts at enriching heterotrophic *Leptothrix* and *Sphaerotilus* spp. from LD iron mats also yielded few results, confirming other studies that the heterotrophic species of *Leptothrix* or *Sphaerotilus* are not common members of microbial iron mat communities [1], [12]. Our working hypothesis had been that *L*. *ochracea* might be more closely related to another family of *Betaproteobacteria*, the *Gallionellaceae*, which consist of known chemolithotrophic FeOB that are found in freshwater Fe-oxidizing communities. The genera, from this family (*Sideroxydans* and *Gallionella*) have been shown to be obligate, lithotrophic FeOB [1], [46], [47]; furthermore, *G*. *ferruginea* produces an Fe-oxide encrusted extracellular stalk. Recent community diversity studies [37], [48]–[50] of microbial iron mat communities similar to the one at LD have found that significant fractions (10–25%) of SSU rRNA clones clustered within the *Gallionellaceae*. Most of these mats were reported to have abundant *L*. *ochracea* type sheaths associated with them, as opposed to the stalks of *G*. *ferruginea*; however only a small fraction (0–3%) of the clones were related to *Leptothrix* spp. This contrasts to the results reported here, where 5% of pyrotagged community from the bulk mat and 40% of the clones from the actively growing periphery of the mat belonged to the *Leptothrix* clade. While members of the *Gallionellaceae* were present in LD iron mats, they only made up 2.5% of the pyrotagged library, and 4.3% of the clone library (**Figure S2**).

A previous model suggested that *L*. *ochracea* was most active at the periphery of iron mats where it contributed to the accretion of new mat [1]. The evidence obtained with the use of the Lepto175 FISH probe supports this model. Analysis of the actively accreting portion of the LD mat indicated that up to 32% of the cells belonged to *L*. *ochracea* but only 9% of the sheaths actually contain cells. These observations suggests that despite sheathed cells being relatively rare in the bulk mat, they still make up a significant percentage of the total population in the mat periphery, where they contribute substantially to biotic iron oxidation and to new mat formation. It is likely that other FeOB, e.g. *Sideroxydans* spp., colonize the matrix of sheaths left behind and continue to oxidize Fe, and that older mat becomes host to other bacterial populations as well, including Fe-reducing bacteria (insert Emerson, field studies AEM reference here). The complexities of these interactions remain to be worked out; however a growing array of tools should assist in developing a more detailed understanding of how different microbial populations interact and control the processes occurring in these Fe-dominated ecosystems.

### Detecting rarely observed sequences

Single cell-based techniques have proven effective at both identifying dominant members of microbial ecosystems that have resisted cultivation, and demonstrating that numerically minor members of a community may have a significant impact on ecosystem function [51]. In this case, we have taken advantage of unique morphological characteristics of *L*. *ochracea* to use FACS to isolate single cells or filaments of cells. In general, isolation of cells using FACS has focused on planktonic cells that grow predominantly in the liquid phase, rather than in consolidated microbial flocs or mats.

Because *L*. *ochracea* grows as filaments of cells, it provides a good signature for FACS sorting. For example, *L. ochracea*, *L. cholodnii* and *S. natans* filaments have a distinctively higher relative florescence and a higher relative side scatter than single cells (**Figure S1;** [52], [53]). The advent of MDA technology for amplification of single cells makes it possible to acquire enough genomic material for sequencing of specific genes, in this case the SSU rRNA and the gyrB gene, and ultimately sequencing the entire genome. Fortuitously, the presence of the sheath, or the Fe-oxyhydroxides that encrust it, do not appear to preclude either cell sorting or WGA, although it is possible that the presence of the sheath may have reduced the efficiency of the process, which could explain the low overall recovery of *L*. *ochracea*-type cells. It is also interesting to note that one of the SAGs we acquired was closely related to the filamentous, methane-oxidizing bacterium, (*Crenothrix polyspora* [44]; SCGC AAA018-I2), suggesting this approach could be used to target other filamentous bacteria.

### Microdiversity of *L*. *ochracea*

Analysis of unique SSU rRNA gene sequences that cluster together with a >99% SSU rRNA gene sequence similarity can reveal the presence of microdiversity within a species and help delineate fine-scale patterns of community composition [54]. The large amount of information generated by pyrosequencing makes it possible to begin to assess population diversity of known ribotypes in complex communities. Analysis of the pyrosequencing data set and the clone library revealed up to 67 different individual *L*. *ochracea* ribotypes and 5 different ribotypes, respectively in the LD Fe-mats. Caution must be taken in interpreting these results due to pyrosequencing artifacts [55]; however, the sheer number of identical, or closely related clones, provides evidence for substantial populations of very closely related, and possibly, clonal ribotypes. It is also possible that minor sequence variation between multiple copies of the SSU rRNA gene within an individual organism could account for some of the observed microdiversity (see, for example, Acinas et al. [54]), the genome of the phylogenetically related *L*. *cholodnii* has only 2 SSU rRNA copies that are 100% identical to one another. Most notably the pyrosequencing data set contained a dominant *L*. *ochracea* ribotype that was identical to the *L*. *ochracea* SAG, as well as being abundant in the clone library (**Figure 4**). This is corroborative evidence that this ribotype was one of the most abundant individual ribotypes in the Fe-mat and indicates it may play a significant role in Fe-oxidation. Other studies have shown that individual ribotypes are often thought to adapt to specific resources or environmental conditions [56], [57], and it also known that spatial and temporal resource partitioning can select for specific ribotypes [58]. It is unknown why one particular ribotype of *L*. *ochracea* appears dominant in the LD iron mat, but it will be interesting to determine the temporal and spatial dynamics of *L*. *ochracea* ribotypes in other iron mats.

### Physiological implications

The findings that *L*. *ochracea* belongs to the *Leptothrix*/*Sphaerotilus* group leaves open the question of its physiology. *L*. *ochracea* cells must excrete a large amount of Fe-oxyhydroxide coated sheath, implying this organism oxidizes large amounts of Fe(II) relative to the cell biomass that is produced. This is consistent with the requirements of a cell that uses Fe(II) as an energy source, since the thermodynamics of Fe(II) oxidation are poor [59], [60]. This contrasts with the known members of the *Leptothrix*/*Spaerotilus* group that are heterotrophic, and whose sheaths are normally filled with cells. The benefit they get from either Fe(II) or Mn(II) oxidation remains unknown. *S*. *natans* is typically thought of as an organism that grows in organic rich environments, e.g. sewage sludge [4], [23]; while other *Leptothrix* spp are often found in freshwater wetlands [45], [61], but not necessarily associated with chalybeate waters. While *L*. *ochracea* has yet to be obtained in pure culture, enrichment studies using flow-through laboratory microcosms have shown that it requires significant amounts of Fe(II) to grow, and may also require natural waters from its habitat [9], [10], [12] Fleming unpublished results). Nonetheless, attempts to enrich or isolate *L*. *ochracea* using static gradient methods or fed batch cultures typically used to isolate *Gallionella* spp. or *Sideroxydans* spp. [62] have not shown success (Emerson, unpublished results). From the single cell analysis done here, we have identified a SAG from *L*. *ochracea*, and this has been submitted for whole genome sequencing. The genome sequence coupled with further ecological and physiological investigations will hopefully establish the true metabolism of this enigmatic microbe.

## Supporting Information

Figure S1 Flow cytometry dot plot of the Fe-oxidizing mat sample stained with SYTO-9 (A) and excited with a 488 nm laser. Displayed are side scatter (SSC) and green fluorescence (FL1) signals. Region R3 was used as the sorting gate. Phase contrast (B) and epifluorescence micrographs of the sorted material (C). Mat samples for single cell genomics were also collected from the margins of the growing Fe-mats and filtered to enrich for filamentous sheaths. Prior to single cell sorting, samples were confirmed to contain between 6–12% ensheathed cells. The high relative SYTO-9 (nucleic acid) fluorescence and high relative side scatter settings on the cell sorter were used to select for a size range consistent with filaments of cells with the approximate size range of *L*. *ochracea*. These cytometer settings were tested previously with ensheathed *L*. *cholodonii* SP-6 cells and observed to select for ensheathed cells. With Fe-mat samples the empty Fe-oxide encrusted sheaths did not fluoresce, nor did they appear to interfere with the operation of the cell sorter. Scale bar on the micrographs is 10 µm.
(TIF)

Figure S2 Neighbor-joining tree of a partial SSU rRNA gene sequence from non-chimeric clones and single amplified genomes of bacteria. The tree was generated using the ARB neighbor joining package with Jukes-Cantor correction. Percentages represent the bootstrap values at each node for 1000 replicate trees and percentages less than 50% are not shown. Originally, sequences were inserted by parsimony into the Silva SSURef NR 99% release 102, (Feb 13, 2010) database and used to assign phyla, orders, classes, families or genus. The number of clones and SAGs (respectively) are listed for each grouping and 4 cultivated *Leptothrix*/*Sphaerotilus* sequences (*) were included as a reference (D16214, L33975, CP001013, Z18534).
(TIF)

Figure S3 Stringency of *L*. *ochracea* specific FISH probe, Lepto175 to *L*. *ochracea* cells present in LD mat samples collected August 2008 (closed symbols) and *O*. *thiooxydans* cells grown in R2A (open circles (**check a**)) or caso- agar (open squares (**check b**)). The relative fluorescence was normalized to hybridizations performed at 5% or 0% formamide. Hybridizations on LD mats were performed on 3 separate occasions using the same conditions and *O*. *thiooxygens* cells were both tested with the Lepto175 probe on the same day as the LD mat with closed triangles. Background was ≤ than 20% of relative fluorescence.
(TIF)

Table S1 The ability of FISH probes to bind to select species of the *Betaproteobacteria.*
(DOC)

File S1 Information regarding additional materials and methods used to generate SSU rRNA gene sequence clone libraries, to analyze the sequence reads, develop the Lepto175 and to perform FISH on Lakeside Drive iron-mat samples.
(DOC)
