# Supplementary figures and images for: What's New Is Old: Resolving the Identity of Leptothrix ochracea Using Single Cell Genomics, Pyrosequencing and FISH
Source: PLoS One. 2011 Mar 17;6(3):e17769. doi: 10.1371/journal.pone.0017769 (PMC3060100; doi:10.1371/journal.pone.0017769)

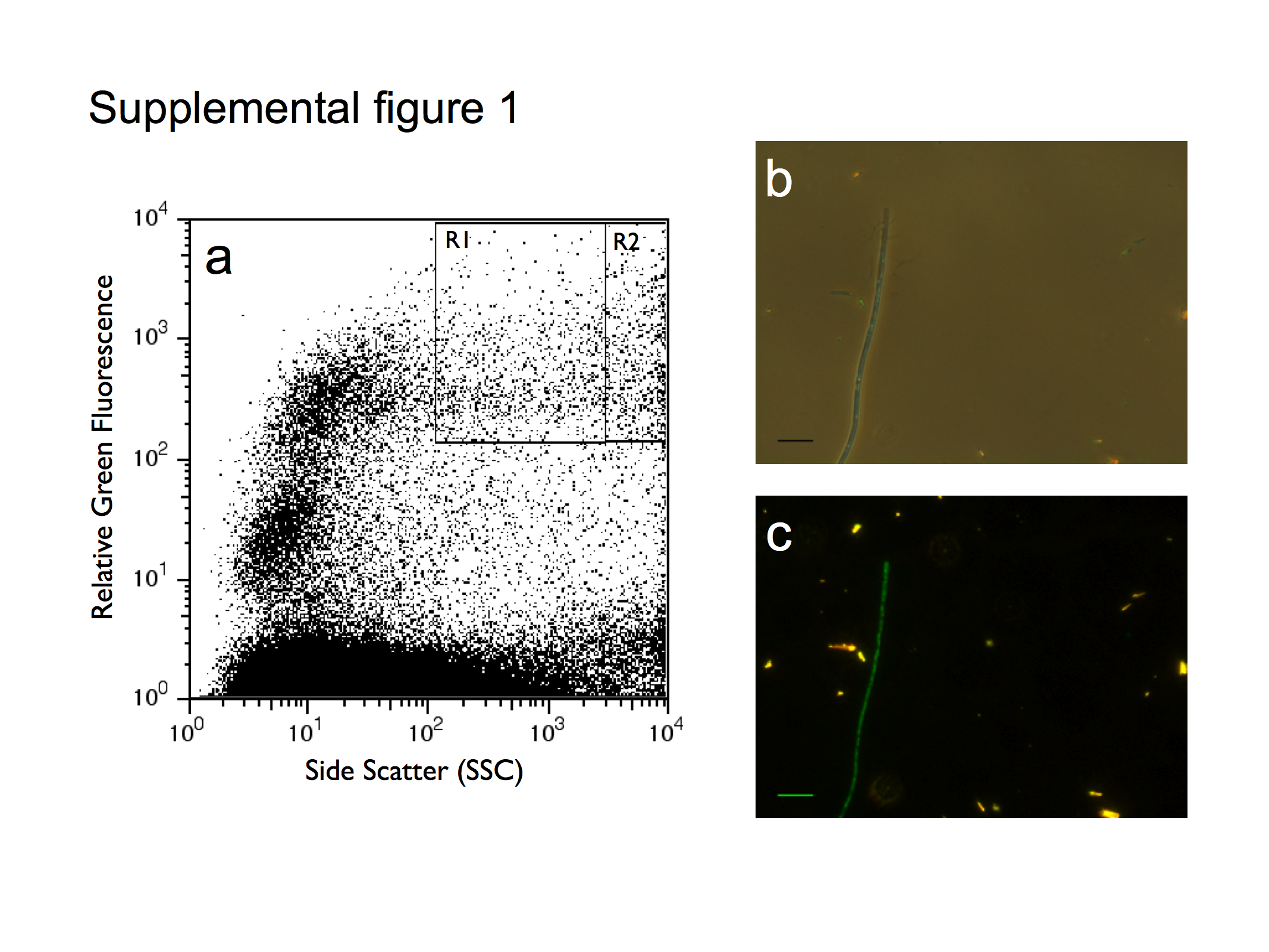

Supplement: Figure S1 — Flow cytometry dot plot of the Fe-oxidizing mat sample stained with SYTO-9 (A) and excited with a 488 nm laser. Displayed are side scatter (SSC) and green fluorescence (FL1) signals. Region R3 was used as the sorting gate. Phase contrast (B) and epifluorescence micrographs of the sorted material (C). Mat samples for single cell genomics were also collected from the margins of the growing Fe-mats and filtered to enrich for filamentous sheaths. Prior to single cell sorting, samples were confirmed to contain between 6–12% ensheathed cells. The high relative SYTO-9 (nucleic acid) fluorescence and high relative side scatter settings on the cell sorter were used to select for a size range consistent with filaments of cells with the approximate size range of L. ochracea. These cytometer settings were tested previously with ensheathed L. cholodonii SP-6 cells and observed to select for ensheathed cells. With Fe-mat samples the empty Fe-oxide encrusted sheaths did not fluoresce, nor did they appear to interfere with the operation of the cell sorter. Scale bar on the micrographs is 10 µm. (TIF) [file pone.0017769.s001.tif]

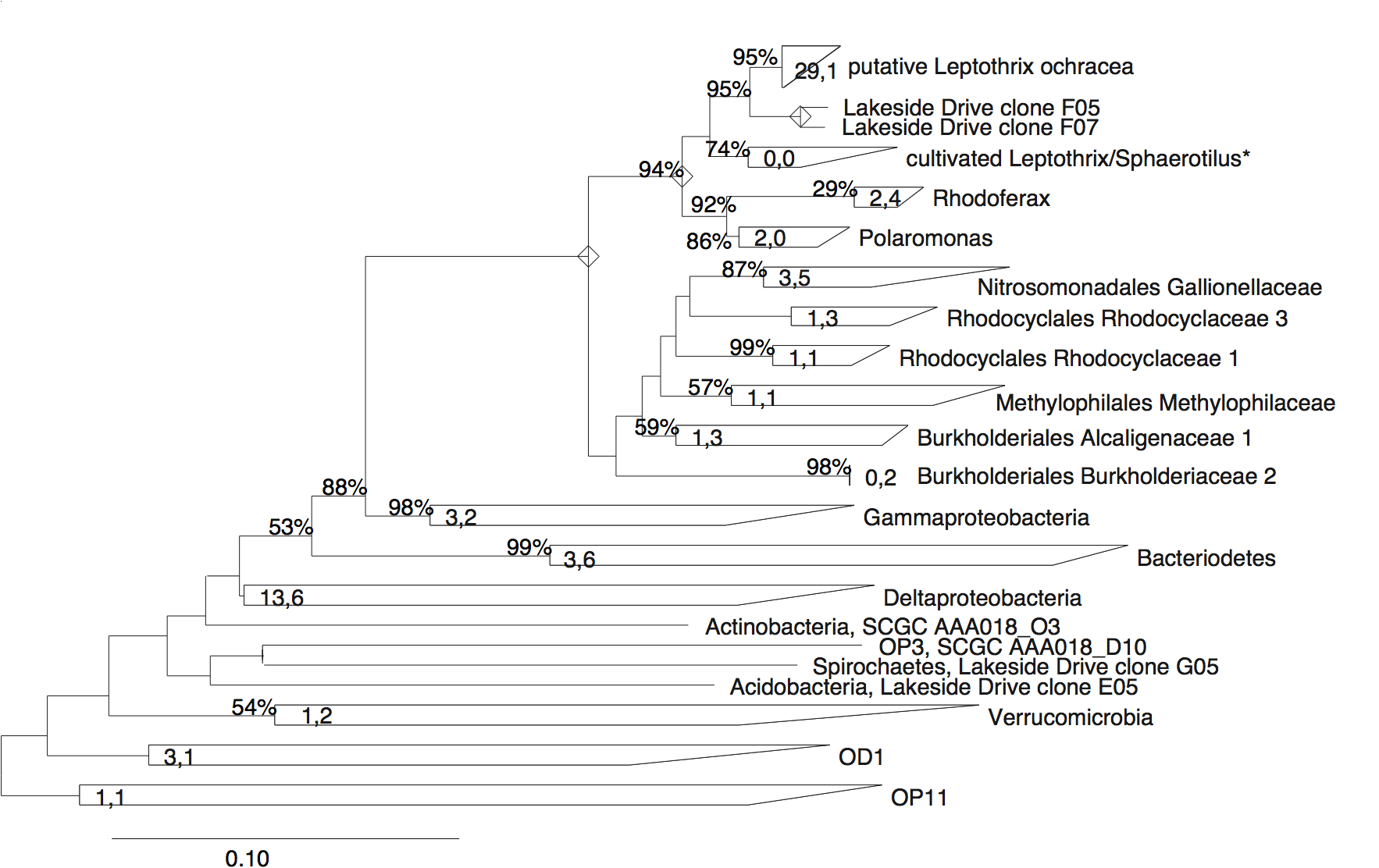

Supplement: Figure S2 — Neighbor-joining tree of a partial SSU rRNA gene sequence from non-chimeric clones and single amplified genomes of bacteria. The tree was generated using the ARB neighbor joining package with Jukes-Cantor correction. Percentages represent the bootstrap values at each node for 1000 replicate trees and percentages less than 50% are not shown. Originally, sequences were inserted by parsimony into the Silva SSURef NR 99% release 102, (Feb 13, 2010) database and used to assign phyla, orders, classes, families or genus. The number of clones and SAGs (respectively) are listed for each grouping and 4 cultivated Leptothrix/Sphaerotilus sequences (*) were included as a reference (D16214, L33975, CP001013, Z18534). (TIF) [file pone.0017769.s002.tif]
