# Supplementary material for: What's New Is Old: Resolving the Identity of Leptothrix ochracea Using Single Cell Genomics, Pyrosequencing and FISH
Source: PLoS One. 2011 Mar 17;6(3):e17769. doi: 10.1371/journal.pone.0017769 (PMC3060100; doi:10.1371/journal.pone.0017769)
Supplement: Table S1 — The ability of FISH probes to bind to select species of the Betaproteobacteria. (DOC) [file pone.0017769.s004.doc]

**Table S1.** Ability of FISH probes to bind to select species of the *Betaproteobacteria*.

|  | | | | | | | |
| --- | --- | --- | --- | --- | --- | --- | --- |
| **Organism** | **Culture or Genomic DNA collection** | **FISH Probe (ProbeBase designation)5** | | | | | |
|  |  | **Lepto1751** | **PS-12** | **Sna 23a (SNA)3** | **LDI3** | **PSP-62** | **Beta1 (BONE)4** |
| *Leptothrix ochracea* | SCGC 00018 L12 | + | -6 | + | - | - | + |
| *Leptothrix cholodnii* SP-6 | ATCC 51168 | - | + | + | - | + | + |
| *Leptothrix discophora* SS*-*1 | ATCC 43182 | - | + | - | + | - | + |
| *Leptothrix mobilis* Feox-1 | DSM 10617 | - | -6 | + | - | + | + |
| *Sphaerotilus natans* Kützing | DSM 6575 | - | -6 | + | - | - | + |
| *Ottowia thiooxydans* K11 | DSM 14619 | + | - | - | -7 | - | + |
| ^1^this study, ^2^[1], ^3^[2], ^4^[3] ^5^[4] ^6^+ with 2 bp mismatch, ^7^+ with 1 bp mismatch | | | | | | | |

1. Siering P, Ghiorse WC (1997) Development and application of 16S rRNA-targeted probes for detection of iron-and manganese-oxidizing sheathed bacteria in environmental samples. Appl Environ Microbiol 63: 644-651.

2. Wagner M, Erhart R, Manz W, Amann R, Lemmer H et al. (1994) Development of an rRNA-targeted oligonucleotide probe specific for the genus *Acinetobacter* and its application for in situ monitoring in activated sludge. Appl Environ Microbiol 60: 792-800.

3. Amann R, Snaidr J, Wagner M, Ludwig W, Schleifer KH (1996) *In* *situ* visualization of high genetic diversity in a natural microbial community. J Bacteriol 178: 3496-3500.

4. Loy A, Maixner F, Wagner M, Horn M (2007) probeBase--an online resource for rRNA-targeted oligonucleotide probes: new features 2007. Nucleic Acids Res 35: 800-804.
