# Supplementary material for: What's New Is Old: Resolving the Identity of Leptothrix ochracea Using Single Cell Genomics, Pyrosequencing and FISH
Source: PLoS One. 2011 Mar 17;6(3):e17769. doi: 10.1371/journal.pone.0017769 (PMC3060100; doi:10.1371/journal.pone.0017769)
Supplement: File S1 — Information regarding additional materials and methods used to generate SSU rRNA gene sequence clone libraries, to analyze the sequence reads, develop the Lepto175 and to perform FISH on Lakeside Drive iron-mat samples. (DOC) [file pone.0017769.s005.doc]

SSU rDNA clone library

The DNA was extracted from the entire filter using the PowerWater DNA isolation kit (MoBio) per the manufacturers instructions. Total DNA was quantified on a NanoDrop Spectrophotometer ND-1000 (Thermo Scientific). Amplification of the SSU rRNA gene sequence was performed with the Takara ExTaq (per the manufacturer’s recommended concentrations). The SSU rDNA gene was amplified from the extracted DNA using universal bacterial primers 27F and 1492R (**Table 1**) The amplification was as follows: an initial denaturation at 94 ℃ for 10 min; 30 cycles of 94 ℃ for 1 min, 50 ℃ for 1 min, and 72 ℃ for 2 min; and a final extension at 72 for 10 min. Amplified DNA was cleaned up using a Qiagen QIAQuick PCR clean up kit and cloned by using the TOPO TA cloning kit (Invitrogen) following the manufacture's protocol. Clones containing plasmids and inserts were grown in LB with Kanamycin (20 μg mL-1), concentrated and transferred to a 96 well microtiter plate. Plasmids were isolated and sequenced at Beckman Coulter Genomics, Danvers, MA, using the 27F and 907R primers for partial sequences (**Table 1**).

1

2

3

4

5

6

7

8

9

10

11

12

13

14

15

16

17

18

19

20

21

22

23

24

25

Sequence analysis

Sequences were analyzed and edited with Sequencher ([http://www.genecodes.com](http://www.genecodes.com/)/) and Genious package [1]. Any chimeric sequences were detected using Bellerophon [2] and subsequently deleted from the data set. High quality sequences were aligned using the Silva aligner ([www.arb-silva.de](http://www.silva.de/), March 23, 2010) and imported into the ARB sequence analysis program. Neighbor Joining trees of the SSU rRNA gene sequences from SAGs and clones were generated using the Jukes-Cantor method using the ARB program [3] and included several reference sequences. The aligned full length SSU rRNA gene sequence from SAG SCGC AAA0018-L12 and several LD clones were used to generate a phylogenetic tree based on the neighbor-joining, maximum-likelihood, and maximum parsimony evolutionary models.

The pyrosequencing reads obtained from the bulk mat sample were exhaustively filtered and checked by using the recently described pipeline PyroTagger [4] that uses high stringency quality-based trimming in order to remove short and low quality reads and dereplications associated with the 454 pyrosequencing technology that can lead to artificial inflation of diversity estimates [5]. Those 454 reads that, at a given read length, had ≥ 3% of bases with Phred values < 27 (0.2 % per-base error probability) were removed from the analysis [4]. Then, a clustering threshold at 99% was used with the resulting high quality pyrosequencing reads. These 454 reads of the bacterial hypervariable region V4 of the SSU rRNA gene sequences were used to build a custom database for BLASTn similarity searches using as query those SSU rRNA gene sequences detected by clone library and SCGs that displayed a unique ribotype in the V4 region (SAG AAA018 L12 and the clones LD H03, D07, E03, and A05). The stand-alone BLAST 2.2.22+ package (ftp://ftp.ncbi.nlm.nih.gov/blast/) was used to perform the analysis. First, the custom database was built by using the BLAST command line application of the user manual. Then, the BLASTn searches were performed for detecting those pyrosequencing reads that displayed ≥99% similarity with the V4 region of the query sequences by using the following parameters: -p blastn -perc_identity 99. The pyrosequencing reads matching the BLAST search parameters were aligned with MUSCLE software [6] implemented in Geneious package ([http://www.geneious.com](http://www.geneious.com/)) and then the different clusters of sequences with unique ribotypes were detected from the nucleotide alignment.

26

27

28

29

30

31

32

33

34

35

36

37

38

39

40

41

42

43

44

45

46

47

48

49

50

Development of Lepto175 probe.

The Lepto175 probe (**Table 1**) was designed (using the ARB software program; Ludwig et al., 2004) to target a grouping of SSU rDNA gene sequence clones and the single SAG SCGC AAA0018_L12. Samples were hybridized with the following probes EUB338-cy3 and NON338-cy3 and BET42a-cy3 were obtained from Integrated DNA Technologies, IDT. GAM42a-Fl, PS-1-cy3 and Lepto-175-cy3 synthesized by Eurofins MWG Operon (Huntsville AL), (**Table 1**) and stored at -20 ℃.

Preparation of cultures and samples for FISH

For FISH, mat samples from either LD, or cultivated control strains were fixed at 4 ℃ in 2.5% paraformaldehyde (final conc.; per Fuchs et al. [7]) for 90 min. After fixation samples were rinsed twice with PBS via centrifugation at 2150 x g in a Eppendorf 5418 microfuge, resuspended in 1:1 PBS: Molecular Biology Grade Ethanol, Sigma and stored at -20 ℃ until use.

For control strains, *Leptothrix* *cholodnii* SP-6 (51168) was obtained from ATCC and grown on ATCC medium 1917 (MSVP) lacking MnCl_2_. Acquired from DSMZ, *Sphaerotilus* *natans* (6575) was grown on DSMZ medium 51 (Beef extract agar slants with a tap water overlay). *Ottowia* *thioxydans* (14619) was also obtained from DSMZ and was cultivated on R2A agarose (DSMZ medium 830) slants with either autoclaved tap water or casoliquid medium (DSMZ medium 220) overlay. *E***.** *coli* from Invitrogen TOP10 cells was grown on LB medium. These control strains were incubated at 30 ℃, harvested during exponential phase and preserved for FISH.

FISH

All samples and control strains were air dried on ClearCell slides with 7 mm diameter wells (ER-279W-2; ThermoScientific). Dried samples were initially incubated for one hour at 46 ℃ in standard hybridization buffer [7] that included the appropriate formamide concentration (between 0% and 55%; Sigma) and several blocking reagents (Denhardt’s solution (3.3x), *E*. *coli* RNA (Ambion) and Sheared Salmon Sperm (Ambion**)).** Samples were hybridized with the appropriate probe per Fuchs et al., [7] then stained with DAPI for 5 min, rinsed with water and allowed to dry. A 4:1 mix of Citifluor and Vectashield (VectorLabs) was added to the sample and slides were sealed with cover glass (Gold seal)

51

52

53

54

55

56

57

58

59

60

61

62

63

64

65

66

67

68

69

70

71

72

73

74

75

For FISH samples the probe binding stringency of Lepto175 and PS-1 was assessed by quantifying the relative fluorescences. From samples hybridized at each formamide concentration a minimum of four epifluorescent images were captured using an Olympus BX60 microscope. The maximum gray value from cross sectional plot profile from at least 32 fluorescently labeled cells was measured to calculate the average maximum fluorescent value generated at each formamide concentration. The relative fluorescence was then calculated from the average maximum fluorescent value of each image divided by the average maximum fluorescence of the least stringent condition (0 % or 5 %).

**Microscopy**

To calculate the total percent of sheaths filled with cells, images of Syto-13 stained *L. ochracea* sheaths/cells were taken at 200x. The recorded images were imported into ImageJ where the entire sheath or the cell filament was traced using a Wacom Intuos 3 PTK-640 pen pad. For each image (n = 9) the percentage difference between the cell filaments and the sheaths was used to calculate the percentage of *L*. *ochracea* filled sheaths.

76

77

78

79

80

81

82

83

84

85

86

87

88

89

90

91

92

93

94

95

96

97

98

99

100

The percentage of *L. ochracea* cells in freshly formed LD mats was determined by FISH using six samples collected in 2008 or 2009. Samples (5 uL) were dried on 7 mm Clear Cell slides hybridized with the Lepto175 probe or the EUB338 probe as described above or were stained with Syto13. From each 7mm well, ten fields were imaged and the total number of micro-organisms or *L*. *ochracea* cells were the average number counted in each well, from this number the total percentage of *L*. *ochracea* cells was calculated.

References

1. Drummond AJ, Ashton B, Buxton S, Cheung M, Cooper A et al. Geneious v5.1. Available: [http://www.geneious.com](http://www.geneious.com/) via the Internet.

2. Huber T, Faulkner G, Hugenholtz P (2004) Bellerophon; a program to detect chimeric sequences in multiple sequence alignments. Bioinformatics 20: 2317-2319.

3. Ludwig W, Strunk O, Westram R, Richter L, Meier H et al. (2004) ARB: a software environment for sequence data. Nucleic Acids Res 32: 1363-1371.

4. Kunin V, Hugenholtz P (2010) PyroTagger: a fast, accurate pipelinde for analysis for analysis of rRNA amplicon pyrosequencing data. The Open Journal 1: 1-8.

5. Kunin V, Engelbrektson A, Ochman H, Hugenholtz P (2010) Wrinkles in the rare biosphere: pyrosequencing errors can lead to artificial inflation of diversity estimates. Environ Microbiol 12: 118-123.

101

102

103

104

105

106

107

108

109

110

111

112

113

114

115

116

117

118

119

120

121

122

123

124

125

6. Edgar RC (2004) MUSCLE: multiple sequence alignment with high accuracy and high throughput. Nucleic Acids Res 32: 1792-1797.

7. Fuchs B, Pernthaler J, Amann R (2007) Single cell identification by fluorescence *in* *situ* hybridization. In: Reddy CA, Beveridge TJ, Breznak JA, Marzluf G, Schmidt TM, Snyder LR, editors. Methods for General and Molecular Microbiology, 3rd Ed. Washington DC: ASM Press. pp. 886-896.
